# Early coronary arterial disease in Sub-Saharan Africa: an emerging challenge for public health

**DOI:** 10.1097/j.pbj.0000000000000293

**Published:** 2025-06-09

**Authors:** Miguel B. A. Vicente, Roger R. Dopico, Luis Mariano, Humberto Morais, Tomás Peralta

**Affiliations:** a Cardiovascular Diagnosis and Intervention Unit, Clínica Girassol, Luanda, Angola; b Coimbra Hospital and University Centre, Coimbra, Portugal; c Department of Haemodynamics and Interventional Cardiology, Carlos J. Finlay Hospital, La Habana, Cuba; d Main Military Hospital, Luanda, Angola; Agostinho Neto University, Luanda, Angola

The earliest investigations into the prevalence of cardiovascular diseases (CVD) in Sub-Saharan Africa (SSA) trace back to the work of British physician Sir Albert Cook at Mulago Hospital, who, between 1897 and 1901, conducted a 3-year retrospective study and found that approximately 3% of admissions were due to CVD, with no records of coronary artery disease (CAD).^1^ Fifty-six years later, a new study identified CAD only in foreigners.^1^ These data reinforced the false notion that Africans were exempt from this condition.

Currently, the World Health Organization and the World Heart Federation indicate that CVD, particularly CAD, are the leading causes of death globally, resulting in around 21 million annual fatalities, which account for 33% of the total. More than 75% of these deaths occur in middle- and low-income countries, especially in SSA, where information on CAD is limited, likely due to a lack of reliable data, diagnostic means, and specialized professionals.^2,3^

Traditionally, CAD affects middle-aged and older individuals in developed countries, being considered premature when it occurs in men and women before the ages of 55 and 65 years, respectively. However, recent evidence shows an alarming rise in CAD among younger populations in SSA, posing a significant challenge to health systems already burdened by infectious diseases (ID), influencing the poor understanding of CAD.^4^

Several isolated studies have reported an increase in premature CAD in SSA, highlighting one conducted in Senegal, which recorded 6.8% of admissions for acute coronary syndrome in individuals younger than 40 years, with smoking being the primary risk factor at 52.4% of cases.^4,5^

This reality is similar in Angola. The retrospective studies conducted by Antunes et al,^6^ who assessed 450 patients with suspected CAD undergoing coronary angiography in an emergency/elective context, and Morais et al,^7^ with 211 patients, who investigated the prevalence of coronary calcifications and associated risk factors in a multiethnic Angolan population, unanimously highlight the increase in CAD, particularly among males in their prime working age, especially those with higher cardiovascular risk factors.

The influence of cardiovascular risk factors is increasingly significant in the prevalence of CAD among Sub-Saharan youth, driven by new socioeconomic behaviors, the adoption of new lifestyles, and rapid urbanization, which promote an epidemiological transition and increase the prevalence of hypertension, diabetes, dyslipidemia, obesity, sedentary behavior, smoking, and pollution. The coexistence of CAD with ID exacerbates the already deficient health management in the region.^1-5^

In light of this scenario, it is imperative that African public health authorities prioritize this emerging issue. Multifaceted measures are necessary, including better characterization of early CAD in SSA, the creation of culturally adapted primary prevention strategies focused on lifestyle changes and risk factor control, as well as strengthening health systems through training of professionals and improving diagnostic infrastructure.
